# The lens capsule significantly affects the viscoelastic properties of the lens as quantified by optical coherence elastography

**DOI:** 10.3389/fbioe.2023.1134086

**Published:** 2023-03-09

**Authors:** Taye Mekonnen, Christian Zevallos-Delgado, Hongqiu Zhang, Manmohan Singh, Salavat R. Aglyamov, Kirill V. Larin

**Affiliations:** ^1^ Department of Biomedical Engineering, University of Houston, Houston, TX, United States; ^2^ Department of Mechanical Engineering, University of Houston, Houston, TX, United States

**Keywords:** lens capsule, viscoelastic properties, lens biomechanical properties, acoustic radiation force, optical coherence elastography (OCE)

## Abstract

The crystalline lens is a transparent, biconvex structure that has its curvature and refractive power modulated to focus light onto the retina. This intrinsic morphological adjustment of the lens to fulfill changing visual demands is achieved by the coordinated interaction between the lens and its suspension system, which includes the lens capsule. Thus, characterizing the influence of the lens capsule on the whole lens’s biomechanical properties is important for understanding the physiological process of accommodation and early diagnosis and treatment of lenticular diseases. In this study, we assessed the viscoelastic properties of the lens using phase-sensitive optical coherence elastography (PhS-OCE) coupled with acoustic radiation force (ARF) excitation. The elastic wave propagation induced by ARF excitation, which was focused on the surface of the lens, was tracked with phase-sensitive optical coherence tomography. Experiments were conducted on eight freshly excised porcine lenses before and after the capsular bag was dissected away. Results showed that the group velocity of the surface elastic wave, V , in the lens with the capsule intact ( V=2.55±0.23 m/s ) was significantly higher (*p* < 0.001) than after the capsule was removed ( V=1.19±0.25 m/s ). Similarly, the viscoelastic assessment using a model that utilizes the dispersion of a surface wave showed that both Young’s modulus, *E*, and shear viscosity coefficient, *η*, of the encapsulated lens ( E=8.14±1.10 kPa,η=0.89±0.093 Pa∙s ) were significantly higher than that of the decapsulated lens ( E=3.10±0.43 kPa,η=0.28±0.021 Pa∙s ). These findings, together with the geometrical change upon removal of the capsule, indicate that the capsule plays a critical role in determining the viscoelastic properties of the crystalline lens.

## 1 Introduction

The primary function of the lens of the eye, along with the cornea, is to focus light onto the retina. Unlike the cornea, the lens has a dynamically modulated curvature and refractive power to produce sharp images of objects at variable distances during a process called accommodation. The mechanism of accommodation is a complex phenomenon, and various theories (Wang and Pierscionek, 2019) were put forward to explain the underlying physiological process. For example, according to Helmholtz’s widely accepted accommodation theory, the lens and its capsule are elastic, and the change in shape and power of the lens involves the capsule transferring the tension produced by the contraction and relaxation of the zonule and ciliary muscles to the lens (von Helmholtz and Southall, 1924; Wang and Pierscionek, 2019). The applied tension deforms the lens, changing the lens curvature, which effectively determines the focal distance of the lens. This intrinsic morphological adjustment of the lens to fulfill changing visual demands has prompted numerous studies on the mechanical properties of the lens and its suspension system, which includes the capsular bag, ciliary muscles, zonules, and choroids (Beers and Van Der Heijde, 1994; Pedrigi et al., 2007; Weeber and van der Heijde, 2007; Ronci et al., 2011; Sharma et al., 2011). In particular, the role of the lens capsule in the accommodative function as well as in cataract surgery, is associated with its biomechanical properties (Huang et al., 2021). The progressive change in capsular mechanical strength due to aging alters the dynamic interaction between the capsule and lens and could lead to changes in the accommodation process. In cataract surgery, a procedure that involves removing the lens content through an opening in the anterior capsule and replacing it with an artificial intraocular lens (IOL), the post-surgical capsular remodeling could have significant biomechanical consequences on not only the capsular matrix but also the lens substance (Berggren et al., 2021). Some other pathological conditions, such as the thinning, rupture, and exfoliation of the anterior lens capsule, could also affect the normal functions of the lens (Irvine, 1940; Liu et al., 2021). Hence, information about the mechanical modulation of the lens with and without the anterior capsular bag is essential to better understand the physiological process of accommodation and to design optimal cataract surgery (Rich and Reilly, 2022).

Over the last few years, the biomechanical properties of the lens have been examined using spinning tests (Burd et al., 2011; Wilde et al., 2012; Reilly et al., 2016), indentation (Weeber et al., 2007; Reilly and Ravi, 2009), Brillouin microscopy (Scarcelli et al., 2011; Ambekar et al., 2020), atomic force microscopy (AFM) (Ziebarth et al., 2011; Avetisov et al., 2021), acoustic techniques (Yoon et al., 2012; 2013; Park et al., 2017), mechanical compression (Won et al., 2015; Cheng et al., 2016), and optical coherence elastography (OCE) (Wu et al., 2015; Wu et al., 2018; Li et al., 2019; Zhang et al., 2019; Ambekar et al., 2020; Chen et al., 2022; Zhang et al., 2022). Using these methods, the lenticular biomechanical properties were assessed as a function of various parameters, including intraocular pressure (Park et al., 2017; Wu et al., 2018) and age and age-related diseases (Scarcelli et al., 2011; Wu et al., 2015; Cheng et al., 2019; Avetisov et al., 2021). On the other hand, typical methods of characterizing capsular biomechanical properties include inflation (Heistand et al., 2005; Avetisov et al., 2020) and uniaxial tensile (Wollensak and Spoerl, 2004) tests on sample fragments. Yet, knowledge of the role of the lens capsule in determining the biomechanical properties of the whole lens is scarce. A microindentation-based mechanical test conducted by applying dynamic displacement waveforms to the lens anterior pole indicated that the lens stiffness decreased significantly after the capsule was removed (Reilly and Ravi, 2009). Despite the significance of the results of this study in providing insight into the potential influence of capsular bag on the mechanical properties of the lens, the method requires cutting the lens to allow assessment of internal stiffness variations, which may disturb the lens structural integrity. In another study, results from spinning tests by Wilde et al. showed that the deformation in the encapsulated lens is less than that in the decapsulated lens for younger subjects and *vice versa* for older subjects (Wilde et al., 2012). This method involves imaging the outline of a lens while it is rotating around its optical axis (typically at 1000 RPM) and quantifying the deformation amplitude induced by centripetal forces. The spinning lens test is advantageous as the lens is subject to minor mechanical disturbances during measurement, but internal stiffness variations cannot be determined directly but rather inferred from axisymmetric finite element (FE) inverse analysis using a neo-Hookean model (Burd et al., 2011). Reilly et al. implemented the inverse FE method to perform mechanical analysis of both the lens and its capsule from a compression test (Reilly and Cleaver, 2017). This method is promising in enabling the assessement of lenses with different shapes, sizes, and mechanical properties, but the assumed model neglects viscous effects and known spatial variations of lenticular biomechanical properties.

In this study, we present a quantitative analysis comparing the viscoelastic properties of porcine lenses with and without a capsule using dynamic wave-based optical coherence elastography (OCE) (Singh et al., 2022; Zvietcovich and Larin, 2022). Here, OCE utilized phase-sensitive optical coherence tomography (PhS-OCT) (Sticker et al., 2001) coupled with an acoustic radiation force (ARF) transducer for non-invasive assessment of tissue mechanical properties at microscale spatial resolution. A microscale and localized tissue displacement induced by focused ARF excitation propagated as an elastic wave and was tracked using a high-resolution PhS-OCT system. The high deformation sensitivity of OCE is important to avoid inducing irreversible hysteresis, which may lead to plastic deformation in some indentation and compression methods. Moreover, small displacements are necessary for clinical applications to ensure adherence to safety limits. Using *ex vivo* porcine lenses, we analyzed the surface elastic wave speed, Young’s modulus, and shear viscosity of the crystalline lens with and without the capsule to shed light on the influence of the capsule on the whole lens biomechanical properties.

## 2 Materials and methods

### 2.1 Porcine samples

Experiments were conducted on eight freshly excised porcine lenses *ex vivo*, both before and after the lens capsule was removed. The whole eye-globes were shipped overnight on ice (Sioux-Preme Packing Co., Sioux Center, IA), and all procedures were performed within 48 h of enucleation. The lenses were mounted on a custom holder.

### 2.2 Optical coherence elastography

**FIGURE 1:**
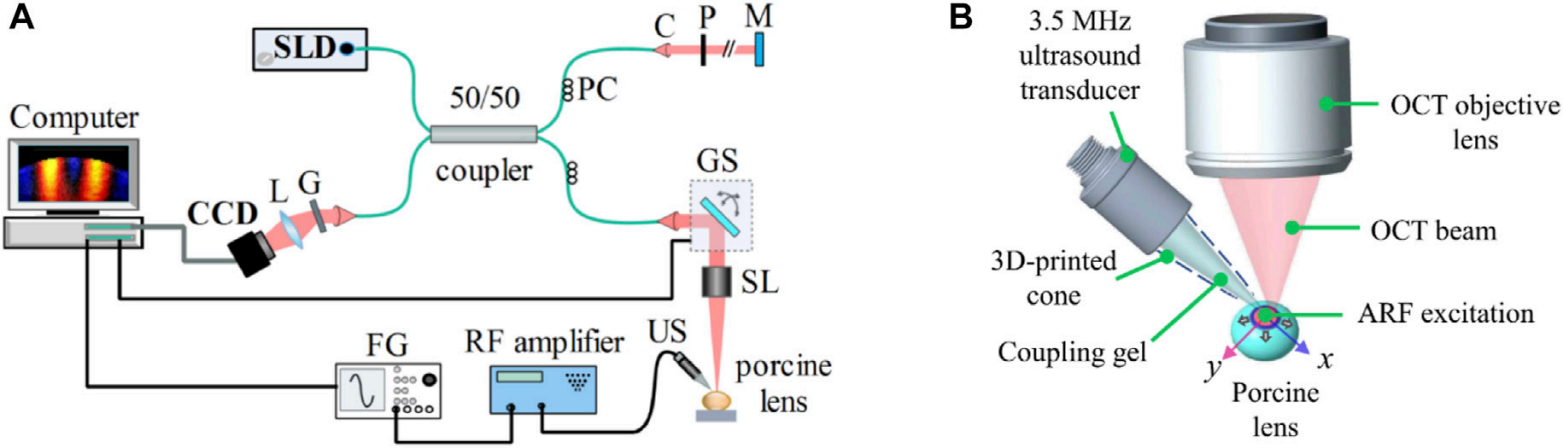
**(A)** Schematic of the experimental setup comprising a phase sensitive spectral domain OCT system for imaging and acoustic radiation force system for excitation. C: collimator, CCD: charge-coupled device (line scan camera), FG: function generator, G: grating, GS: 2D galvo scanner, L: lens, M: reference mirror, P: pinhole, PC: polarization controller, SL: scan lens, SLD: superluminescent diode, US: ultrasound transducer. **(B)** Ultrasound transducer producing acoustic radiation force excitation at the apex of the lens. Propagating elastic waves were imaged and analyzed along the orthogonal *x* and *y* axes.

The OCE system shown in Figure 1A was comprised of a 3.5 MHz ultrasound transducer of focal length ∼19 mm (V382-SU, Olympus Corp., Japan) coupled with a PhS-OCT system that employed a broadband superluminescent diode (S840-B-I-20; Superlum Diodes Ltd., Carrigtwohill, Ireland) operating at 840 nm center wavelength with FWHM of 49 nm as a light source. The axial resolution of the system was ∼9 µm in the air, while the displacement stability and transverse resolution were 0.28 nm and ∼8 μm, respectively. The transducer driving signal, a continuous 3.5 MHz sinusoidal signal modulated by a square pulse of short duration (i.e., 0.5 ms), was generated by a function generator (DG4162, RIGOL Tech, Beijing, China) followed by amplification using an RF power amplifier (1040L, Electronics & Innovation, Ltd., Rochester, NY, United States). The excitation, which was coupled to the lens using ultrasound gel (McKesson Ultrasound Gel Pink, Richmond, VA), was focused roughly on the apex of the anterior surface of the lens, as shown in Figure 1B. M-B mode scans (Wang and Larin, 2014) were performed along orthogonal axes, which are marked as x and y in Figure 1B, intersecting at the excitation point. Each M-mode scan contained 1000 A-lines and was repeated at 251 lateral points (B-scan), covering scan lengths of 7.67 mm and 7.72 mm on the two orthogonal axes. Measurements were conducted at an A-line rate of 25 kHz.

### 2.3 Data processing

The acquired OCE data was processed using MATLAB^®^ R2021a (Mathworks, Inc., Natick, MA, United States). First, the axial phase shift was computed from the temporal A-scans, followed by producing the spatiotemporal map of the elastic wave propagation. Then, the elastic wave group velocity was computed from the spatiotemporal phase map using the ratio of propagation distance to corresponding time (i.e., the slope in the spatiotemporal image) (Zvietcovich and Larin, 2022). This procedure was repeated for the subsurface layers of the lens, and a depth-wise averaging over a thickness of ∼0.4 mm was performed to obtain the mean elastic wave speed for each lens.

### 2.4 Viscoelastic quantification

Group velocity alone may not fully describe the biomechanical properties of lossy media such as tissues (Parker et al., 2018; Zvietcovich and Larin, 2022). Hence, quantifying the viscoelastic properties of the lens (Zhang et al., 2022) would more accurately describe the capsular influence on lenticular biomechanical properties. Elastic waves induced by short-duration ARF pulse, such as the one in this study, are composed of multiple frequencies, and thus, dispersion curves (i.e., phase velocity as a function of frequency) can be produced by spectrally decomposing the elastic wave propagation obtained from OCE measurement data. To this end, a 2D discrete fast Fourier transform (FFT) was applied to the spatiotemporal displacement map to obtain the wavenumber ( k ) *versus* frequency ( $\left.f\right)$ magnitude map (Han et al., 2016; Kijanka and Urban, 2021). Then, the phase velocity-frequency map was produced using the relation $c_{p}=f/k$ . Subsequently, the surface wave dispersion curve was obtained by selecting the maximum intensity for each frequency. To assess the viscoelastic properties of the lens, we applied a rheological Kelvin-Voigt (KV) model in which the complex shear modulus is given by $\mu_{D}=\mu +i\eta \omega$ , where ω=2πf is the angular frequency of vibration and; μ and η are the shear elasticity and shear viscosity moduli, respectively. Given the limited penetration depth of the elastic wave and the free space-tissue boundary for an isolated lens, the detected elastic wave was modeled as a surface wave (Rayleigh wave) (Nenadic et al., 2011; Zhang, 2016). Assuming the lens is a nearly incompressible material, the lens shear wave velocity, $c_{s}$ , and Rayleigh wave velocity, $c_{R},$ are related by $c_{R}/c_{s}=0.95$ . Here, the Rayleigh wave model was used to estimate the viscoelastic properties because the mean thickness (T) of the lens at the measurement regions, i.e., near the apex of the lens (T_encapsulated_ = 4.9 mm; T_decaspulated_ = 4.1 mm), was determined to be greater than the wavelength of the induced elastic wave (λ_encapsulated_ = ∼3.6 mm; λ_decapsulated_ = ∼1.7 mm) at the center frequency of excitation of 706 Hz**.** Therefore, solving a one-dimensional Helmholtz equation, the phase velocity of the elastic wave, $c_{p},$ utilizing the KV model can be computed as (Jin et al., 2020; Liu et al., 2020):

$$c_{p}\left(\omega \right)=0.95\sqrt{\left(\frac{2\left(\mu^{2}+\omega^{2}\eta^{2}\right)}{\rho \left(\mu +\sqrt{\left(\mu^{2}+\omega^{2}\eta^{2}\right)}\right)}\right)}$$ (1)

where $\rho =1183\;kg/m^{3}$ was the lens density (Vilupuru and Glasser, 2001). The shear modulus parameters, i.e., μ and η , were determined by fitting the viscoelastic wave Equation 1 to the OCE-measured surface wave dispersion curve using the iterative Levenberg-Marquardt error optimization algorithm. Assuming an isotropic and homogenous lens, the elastic (Young’s) modulus, *E*, was computed from the shear modulus using $E=2µ\left(1+v\right)$ , with the Poisson’s ratio, v=0.499 .

Furthermore, we assessed the wave amplitude attenuation characteristics using the intensity map in the spatial-frequency domain. The intensity map was produced by applying a 1D FFT on the spatiotemporal map of the wave field. In the spatial-frequency domain, for each lateral position, x , the wave amplitude profile was fitted to the exponential decay function for cylindrical wave ( ${C_{o}/\sqrt{x}∙e}^{-\alpha x}$ ) to estimate the attenuation coefficient, α, at the center frequency of excitation, where *C*_*0*_ is a constant (Zvietcovich and Larin, 2022).

### 2.5 Lenticular morphology

To investigate the relationship between lens morphology and its biomechanical properties, we quantified the lens geometry using a swept source OCT system that was able to capture the whole lens. The system operated at a center wavelength of 1310 nm, bandwidth of 100 nm, imaging depth of over 7 mm (in air), and a sweep rate of 100 kHz. A 3D scan of the whole lens was acquired using this system, and volumetric images were reconstructed using a custom MATLAB^®^ R2021a (Mathworks, Inc., Natick, MA, United States) program. From the volumetric images of the whole lens, geometric parameters such as the equatorial diameter and the sagittal (apical) thickness were quantified (Wang and Pierscionek, 2019). To obtain more accurate geometric parameters, image distortions caused by refraction and the scanning mechanism (non-telecentric) were corrected using the lens refractive index (n_l_ = 1.49) and a 3D non-telecentric scan correction method (Zhao et al., 2010), respectively. Results were statistically analyzed using a *t*-test to assess the significance of variation before and after the removal of the capsule. Also, the repeatability of the experiment was assessed using ANOVA single-factor analysis.

## 3 Results

### 3.1 Elastic wave group velocity

**FIGURE 2:**
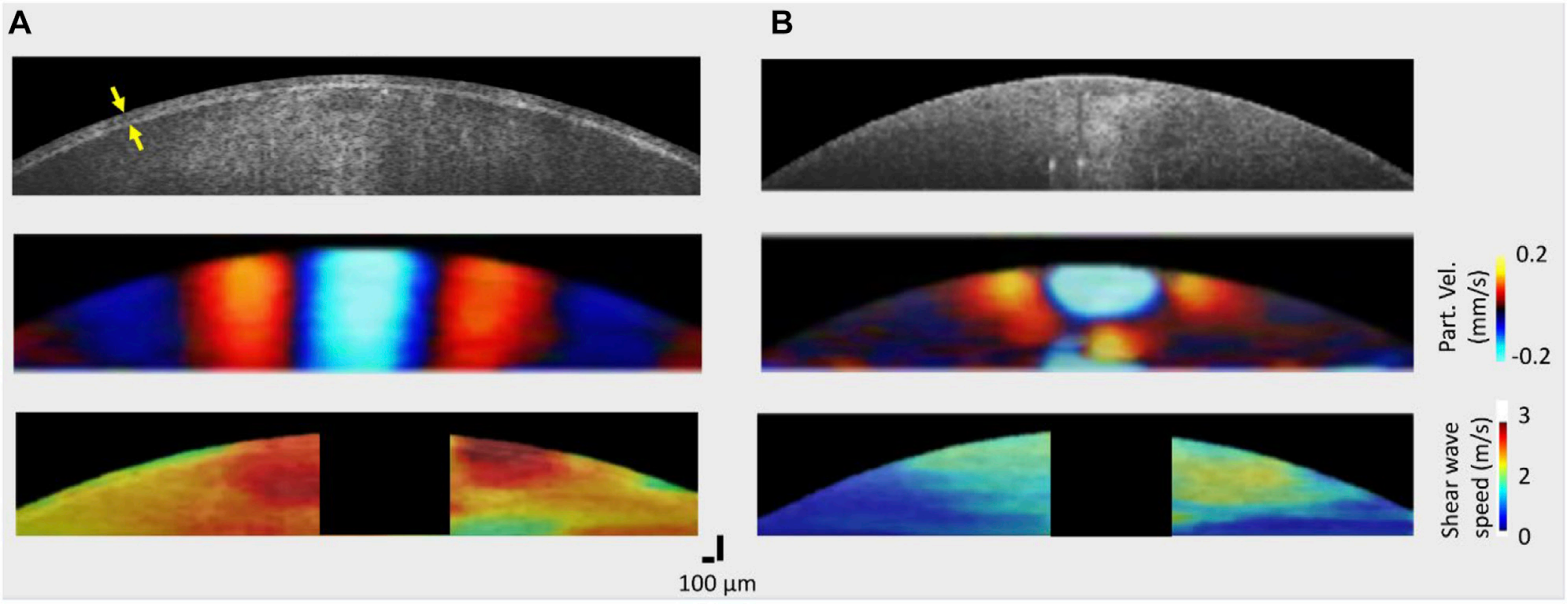
OCT structural images and elastic wave propagation characteristics of a typical porcine lens **(A)** with the capsule intact and **(B)** after the capsule was removed. Top: OCT structural images acquired before (left) and after removal (right) of the capsule; middle: wave propagation snapshots indicating instantaneous particle velocity in an encapsulated (left) and decapsulated (right) lens; bottom: spatial shear wave speed map in the encapsulated (left) and decapsulated (right) lens. The capsule layer is indicated by the yellow arrow in the top left structural image. For all samples, the excitation location was roughly at the apex.

Figure 2 shows the structural images and elastic wave speed characteristics in a typical porcine lens before (left) and after (right) removal of the capsule. In (Figure 2A, top), the capsule with a mean thickness of 58 ± 7 µm can be resolved (as shown by the yellow arrows) in the structural image. Furthermore, the motion snapshot of wave propagation at 2 ms after excitation shows a difference in the wavelength between the encapsulated and decapsulated lens (Figures 2A, B middle): longer in the encapsulated lens than the decapsulated lens. As can be observed from the middle images of Figures 2A, B, the elastic wave propagates further laterally in the encapsulated lens while the wave attenuates faster for the decapsulated lens. The wave attenuation characteristics are presented in the subsequent discussion. Moreover, the bottom row in Figures 2A, B depicts shear wave group speed maps. The wave speed maps indicate that the lens is stiffer with the capsule intact (average speed = 2.55 ± 0.23 m/s) than after the removal of the capsule (average speed = 1.19 ± 0.25 m/s). While there is likely regional variation in the stiffness of the lens, the significant difference in wave speed between nearer and farther regions from the excitation point shown in Figure 2B (bottom) might just be due to the rapid wave attenuation, and low signal to noise ratio in farther regions.

**FIGURE 3:**
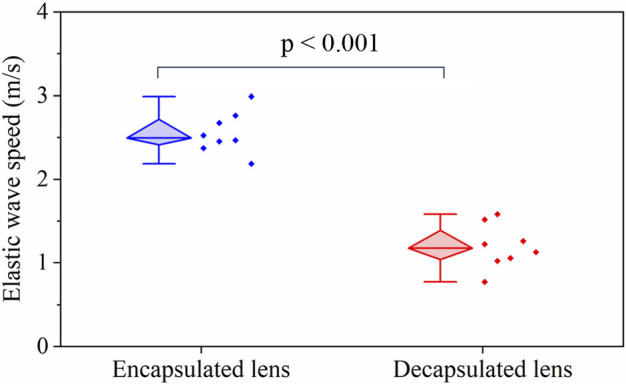
A box-whisker plot and measured data set distribution of the elastic group wave speed in the porcine lens before and after removal of capsule for N = 8 porcine lenses. The horizontal bar in the diamond box corresponds to the median of the data.

Figure 3 shows a box-whisker plot of the mean elastic wave speed in the lens for the two measurement conditions: with and without the capsule. The top and bottom boundaries of the box are the 25th and 75th percentiles, respectively, while the mean is shown by the horizontal bar inside the diamond box. The distribution of the mean wave speed for the two groups is shown by the scatter plots in Figure 3. The mean wave speed with the capsule intact (2.55 ± 0.23 m/s) is approximately twice the value after the capsule was dissected away (1.19 ± 0.25 m/s). Statistical testing by a one-way ANOVA showed no significant intra-group difference in the wave speed for both the capsulated (F (7,26) = 0.58, *p* = 0.75) and decapsulated (F (7,16) = 0.32, *p* = 0.92) states, highlighting the repeatability of the experiment. The inter-group statistical analysis using a student t-test showed that the wave speed was significantly higher with the capsule intact than after dissecting it away (*p* < 0.001).

### 3.2 Lenticular viscoelasticity

**FIGURE 4:**
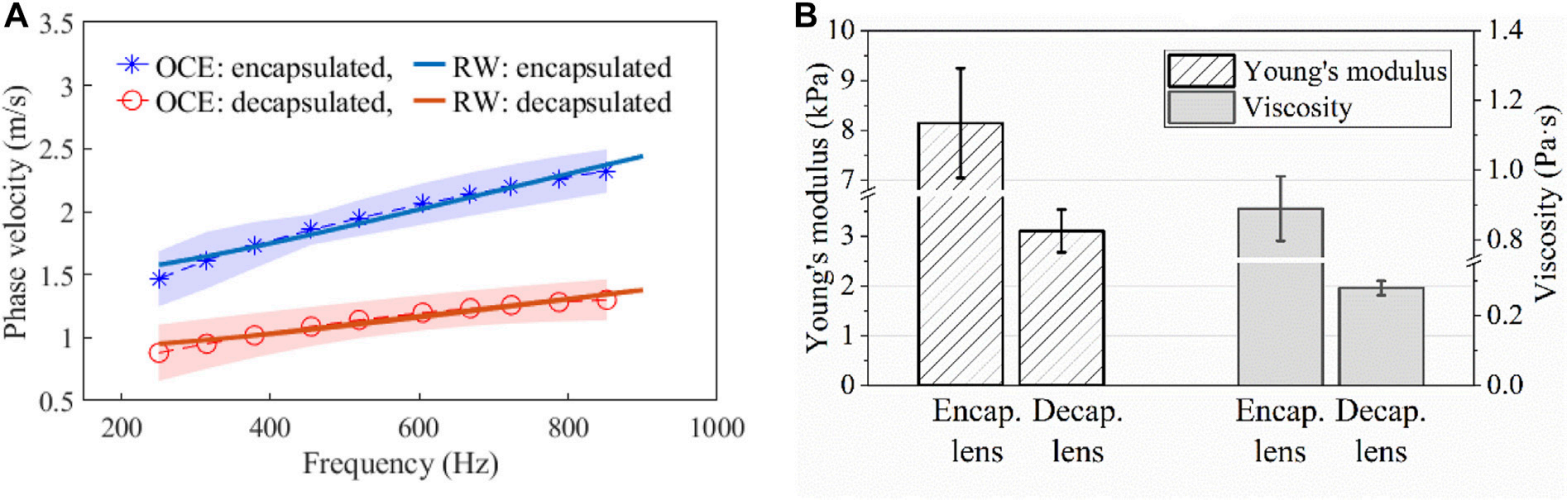
**(A)** Typical elastic wave dispersion curves in the porcine lens before and after capsule removal fitted with Rayleigh wave dispersion equation. The shaded region indicates the error band (standard deviation) of the OCE results. **(B)** Estimated Young’s modulus and viscosity coefficient using Rayleigh wave dispersion equation for encapsulated and decapsulated lens. N = 8 porcine eye lenses.

Figure 4A shows a dispersion curve (i.e., phase velocity as a function of frequency) obtained from an OCE measurement and the Rayleigh surface wave curve fitted to the data. For the selected frequency range, it appears that the rate of change of velocity with frequency is greater for the encapsulated lens. Figure 4B depicts a summary of the viscoelastic properties of the encapsulated and decapsulated lens estimated using the phase velocity dispersion curve fitted to the Rayleigh surface wave model. The Young’s modulus, *E*, and shear viscosity coefficient, *η*, decreased from *E* = 8.14 ± 1.10 kPa and *η* = 0.89 ± 0.09 Pa⋅s in the encapsulated lens to *E* = 3.10 ± 0.43 kPa and *η* = 0.28 ± 0.02 Pa⋅s in the decapsulated lens. The mean Young’s modulus and viscosity coefficient of the decapsulated lens were both significantly lower than that of the encapsulated lens (*p* < 0.001).

**FIGURE 5:**
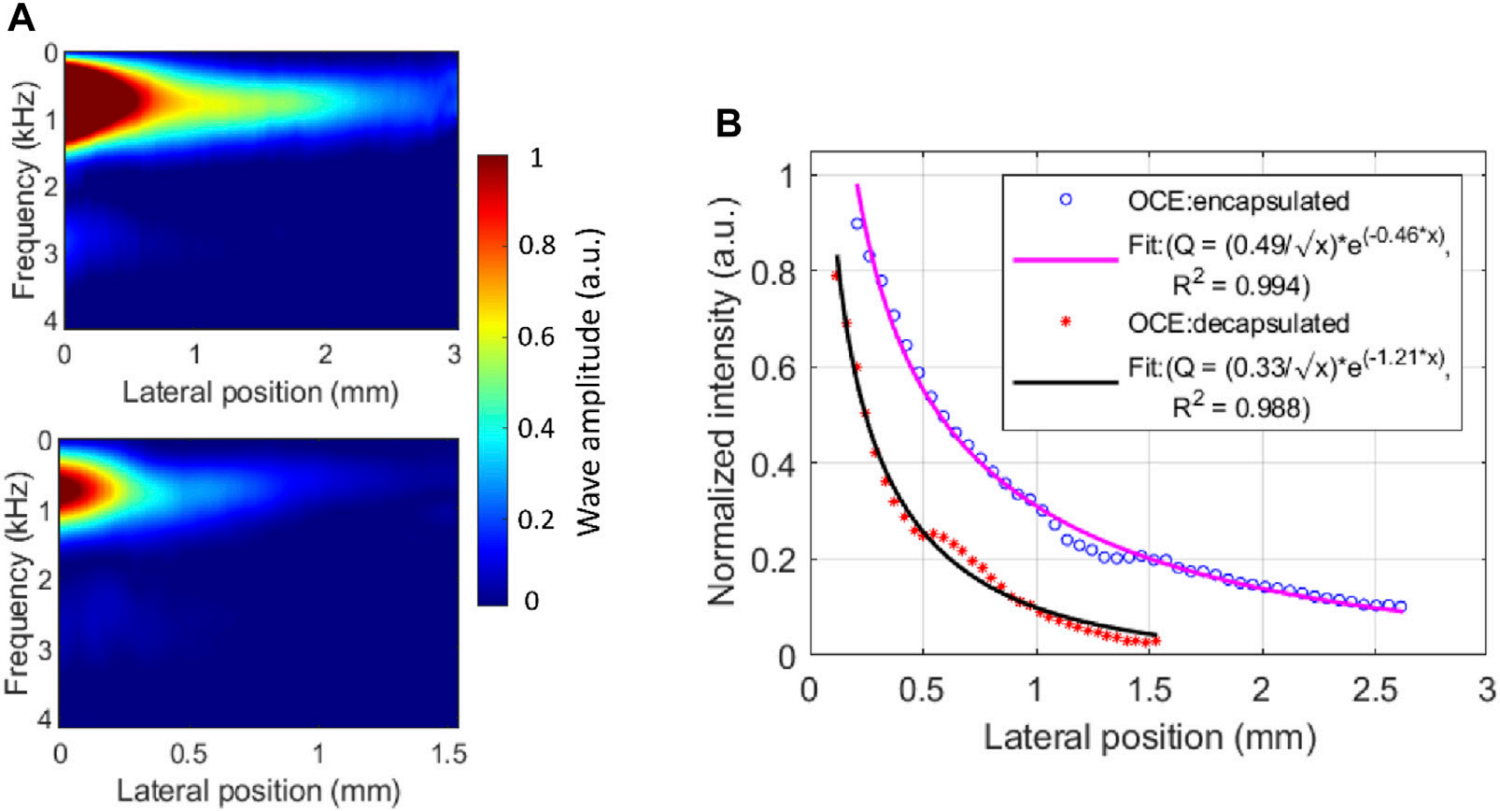
Elastic wave attenuation characteristics in a typical porcine eye lens. **(A)** The energy distribution map of laterally propagating elastic wave in encapsulated (top) and decapsulated (bottom) lens. **(B)** The normalized spatial distribution curve of the peak intensity and the exponential fitting before and after capsule removal. The peak intensity occurred at a center frequency of ∼706 Hz. At the center frequency, the mean attenuation coefficient is higher in the decapsulated lens ( $\alpha =1.21{mm}^{-1})$ than in the encapsulated lens ( $\alpha =0.46\;{mm}^{-1}$ ).

In addition to the change in elastic wave velocity (i.e., dispersion) with frequency, characterizing the amplitude reduction (i.e., attenuation) as the elastic wave propagates through the medium, would provide further insight into the viscoelastic properties of the lens, as we have shown in the cornea previously (Li et al., 2014). Here, we quantified the amplitude attenuation of the elastic wave propagated across the lens in the lateral direction using the wave intensity map in the spatial-frequency domain, as shown in Figure 5A, which was produced by applying a 1D FFT on the spatiotemporal displacement map. Comparing the top and bottom intensity maps in Figure 5A, the wave amplitude dissipates more rapidly in the decapsulated lens than in the encapsulated lens, where ∼80% of wave amplitude attenuated at 1.39 mm and 0.68 mm of propagation, respectively. This difference can be observed in the normalized spatial distribution profile of the peak intensity shown in Figure 5B. From the exponential decay fitting, the attenuation coefficient of the surface wave in the decapsulated lens was found to be roughly twice that of the encapsulated one (ratio = 1.21/0.46 = 2.63) at a center frequency of 706 Hz.

### 3.3 Lenticular morphology

**FIGURE 6:**
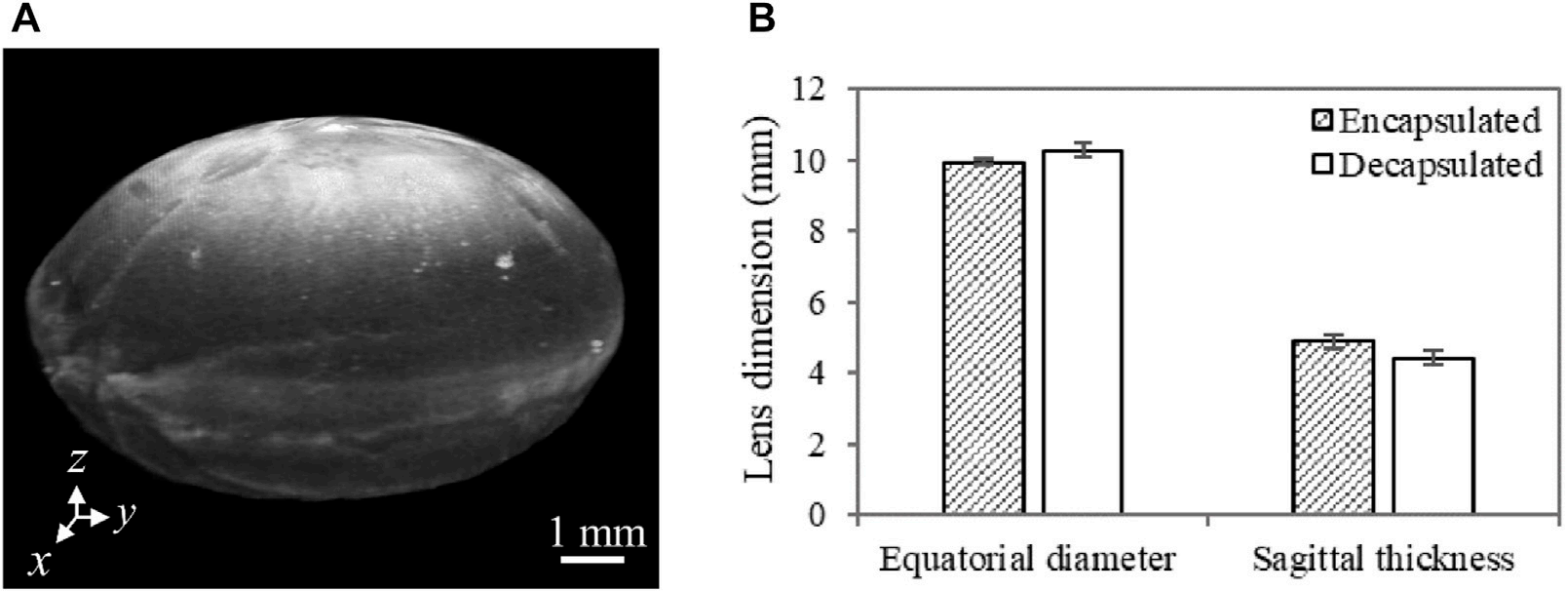
**(A)** A representative 3D OCT image of a dissected porcine lens. The anterior region is facing up (i.e., along the *z*-axis). Lens sagittal thickness represents the maximum thickness along the anterior-posterior direction (along the *z*-axis), while the equatorial diameter stands for the maximum thickness along the *x*-axis or the *y*-axis. **(B)** Porcine lens geometric features characterized by the sagittal thickness and equatorial diameter with and without the capsule. For each sample, multiple cross-sectional images (n = 4) extracted from 3D OCT images were used to quantify the mean geometric features. N = 3 for each group of lenses.

A summary of the lens equatorial diameter and sagittal thickness, quantified using OCT images, is shown in Figure 6B. The results indicate that the lens sagittal thickness is slightly lower while the equatorial diameter is slightly higher after capsule removal. Despite the observed consistency in this trend among all samples, the difference in both geometric features between encapsulated and decapsulated lenses was not statistically significant. However, it is worth noting that the decrease in the sagittal thickness (470 ± 19 µm) was greater than the thickness of the removed capsule (58 ± 10 µm). The increase in equatorial diameter and the decrease in axial/sagittal thickness with the removal of the capsule lead to an increase in the radius of curvature of the lens.

## 4 Discussion

This study aimed to investigate the viscoelastic properties of the porcine lens using ARF-based OCE, and, specifically, to assess the influence of the capsular bag on the biomechanical properties of the lens. A comparison of elastic wave speeds demonstrates that the lens was significantly stiffer with the capsule intact than after the capsule was dissected away (*p* < 0.001), as shown in Figure 3. The viscoelastic properties of the lens quantified using the dispersion of a Rayleigh wave also showed a similar trend of significantly greater Young’s modulus and shear viscosity coefficient in encapsulated lenses compared to their decapsulated counterparts (*p* < 0.001), which is plotted in Figure 4B. Furthermore, the shorter wave propagation distance observed in the decapsulated lenses correlated with a greater magnitude of attenuation coefficient, as plotted in Figure 5A. The wave attenuation coefficient of the decapsulated lenses was roughly twice that of the encapsulated lenses at the measured center frequency, indicating a higher rate of exponential decay in wave amplitude in the decapsulated lens as a function of propagation distance from the excitation position. These results suggest that the lens capsule plays a significant role in determining the mechanical properties of the lens.

The intra-sample correlation assessment of group wave speed using one-way ANOVA indicated the repeatability of the measurements both with (F (7,26) = 0.58, *p* = 0.75) and without (F (7,16) = 0.32, *p* = 0.92) capsule. For the encapsulated lens, the estimated Young’s modulus was 8.14 ± 1.10 kPa and is in good agreement with previous OCE studies conducted on the porcine lens (Zhang et al., 2019; Ambekar et al., 2020; Chen et al., 2022). Despite the difference in the loading frequencies, the change in the viscoelastic properties with the removal of the capsule showed a similar trend to prior studies (Reilly and Ravi, 2009). The encapsulated lens appears to be significantly stiffer and has a higher shear viscosity (*p* < 0.001) relative to the isolated lens matrix, reinforcing the notion that the lens exhibits viscoelastic properties (Schachar et al., 2007). The elastic wave attenuation coefficient and the frequency-dependent phase velocity presented in this study could be important for the mechanical modeling of a lens with and without a capsule during personalized refractive procedures.

From the morphological point of view, the decrease in the sagittal thickness as well as the increase in the equatorial diameter with the removal of the capsule (Figure 6), suggests that the lens relaxes by remodeling its internal structure and does not retain its original shape after the capsule is removed. Thus, the capsule prevents the lens from flowing away, or that the lens is in a compressed (accommodated) state while the capsule is intact. Furthermore, the volume of the crystalline lens, as determined using the discrete integration method described by Marussich et al., showed no significant change (*p* > 0.05) after capsule removal, indicating that the change in morphology is potentially due to the redistribution of the internal tissue structure (Marussich et al., 2015). This morphological change coincides with the decrease in the elastic and viscous moduli, signifying that the capsule plays an important role in maintaining the morphology of the lens. In essence, with its low elasticity a dominant feature, the lens could assume an unwanted shape (e.g., the tendency of flattening or bulging anteriorly/posteriorly) when capsular integrity is compromised (e.g., due to disease or aging) or in the absence of (weakened) capsular support. Normally, alterations in the organization of constituent collagen IV and laminin meshwork and a reduction in the percentage of collagen IV with age could cause a change in capsular structural integrity (Rich and Reilly, 2022). Capsular support may also be compromised due to complications in extracapsular cataract extraction or phacoemulsification procedure (Por and Lavin, 2005).

While this study successfully demonstrated the mechanical interaction between the lens and its capsule, there are a few limitations that could be addressed in future research. First, porcine eyes lack the ability to accommodate and hence, may not be an appropriate model for human eyes. However, the results of the current study can be relevant in assessing the biomechanical properties of eyes with accommodative dysfunction, such as aged human or presbyopic eyes. Second, lens stiffness was characterized based on the propagation of the elastic wave in the selected area, which was at the anterior apex of the lens, mainly due to the low internal optical scattering of the lens substance. Third, it was not possible to discern the stiffness of the thin capsule from the results of the current study, mainly due to the relatively long wavelength of the induced elastic wave.

## 5 Conclusion

The current study highlights the influence of the capsule on the biomechanical properties of the lens as well as demonstrates the capability of the non-contact OCE system to provide a quantitative assessment of lens stiffness as a function of the capsule. Our study suggests that the measurement of the lens and its capsule stiffness as a unit may not reflect only the crystalline lens stiffness, which appears to be significantly influenced by the capsule. Future studies may consider quantifying the spatial anisotropy in the viscoelastic properties of the lens to provide a comprehensive assessment of the significance of the capsule in determining the structural integrity and function of the lens. Furthermore, higher excitation frequencies may assist in increasing elastic contrast and hence, discerning regional variations in lens stiffness, e.g., resolving elasticity gradient in the cortex and nucleus as well as the thin capsular layer.

## Data Availability

The raw data supporting the conclusions of this article will be made available by the authors, without undue reservation.
